# The Duty of the Mother and the Home in the Sexual Education of Children

**Published:** 1910-12

**Authors:** 


### The Duty of the Mother and the Home in the Sexual Education of Children.

Krukenberg (Zeitchr. f. Bekaempfung der Geschlechts Krankheiten) defines the position of the mother and of the home in the matter of sexual education of the young. She denounces the spirit of falsehood and concealment that everywhere pervades the discussion of these subjects in the family. Both school and home are to blame for this system, but of the two the home is more to be censured because it is primarily the responsible party for the proper raising of children. Her conclusions are:

1. A special sexual education is not necessary where there are at home healthy and pure-minded fathers and mothers, who will, as occasion presents, from the very onset tell their children the truth in a manner suited to the age of the child.

2. It must be our aim to educate fathers and mothers in such wise that special enlightenment from other sources will become more and more superfluous.

3. Home education has the following advantages over school education: (a) the parent can, as occasion arises, without undue emphasis, give talks upon these subjects; (b) it can be graduated with each child in accordance with its development; (c) it can prevent false impressions from the very start.

4. The home often counteracts the work of the school by an unnatural prudish secrecy or by jests and laughter and equivocal remarks concerning that which the child has learned in school.

5. The manner of elucidating this subject is often a wrong one. Fanatics in sex education often say too much and lay too much stress upon sexual matters in education. Prolonged repetition of this theme as it occurs in some of the books in sex hygiene, is to be avoided. A short, clear answer is all that is needed; further questioning can be turned aside by a tactful change of subject. If, however, questions have been asked, the reply must be truthful, short and to the point, as if the whole thing were a matter of course.

6. The evil of self-abuse should be fought against in early youth, without giving it a name: (a) by careful observation of the child by the mother; (b) suitable posture during sleep (c) by warning it against injury to its health; (d) by warning against temptation among school companions.

7. Before leaving the home of their parents, young men should be told of the dangers of extra marital sexual intercourse. In this instance a pamphlet or a book will often serve a better purpose than a personal conversation. Personally a few words touching upon their sense of honor, and their responsibility toward their future wife and children will be all that is necessary.

8. Girls who must leave home to earn their living should likewise be duly warned (possibly also by means of a suitable pamphlet).

9. Young girls who remain in the homes of their parents need not be given detailed information concerning venereal diseases, prostitution, etc.

For them it is sufficient to explain:

(a) That menstruation is a necessary process to allow for the expulsion of material, necessary for the development of a new phase of life.

(b) That marriage for money is a sin against nature, a degradation for life.

(c) That the "sowing of wild oats" by men before marriage is the cause of much sickness to wives and to the future offspring.

(d) That good health and cleanliness should be their duty, not only for the sake of their future children, but also, if unmarried, to make them better fitted for their calling in life.

10. Sex education at home does not require much time but only a sane conception of sexual life on the part of the parents and an open, cordial relationship between mother and child. This can be found just as well in the homes of the poor as elsewhere.—*Indian Medical Record, Delhi, India.*

